# Paraplegia of late onset in adolescents with healed childhood caries of dorsal spine: A cause of pressure on the cord and treatment

**DOI:** 10.4103/0019-5413.41871

**Published:** 2008

**Authors:** Rangachari Paravastu

**Affiliations:** Department of Orthopaedics, Osmania Medical College, Hyderabad, Andhra Pradesh, India

**Keywords:** Late onset of paraplegia in adolescents, management, anterior decompression

## Abstract

**Background:**

Paraplegia of late onset in adolescents with caries of dorsal spine is considered to be due to the reactivation of infection. Internal salient at the level of acute kyphotic deformity of the dorsal spine is formed by posterior cartilaginous remains of grossly destroyed vertebral bodies. The author presents a study of eight adolescent patients with paraplegia of late onset associated with severe kyphotic deformity of dorsal spine with observations on the cause of paraplegia, the final neurological outcome following anterior decompression and its prevention.

**Materials and Methods:**

Eight adolescent patients mean age 14.4 yrs 6 males and 2 females with healed childhood caries of dorsal spine, having a mean kyphotic angle of 80° (range 60°–140°) presented with paraplegia of late onset. Of these patients, two had medical research council grade 0 muscle power; four had grade 2 muscle power, and two others had grade 3 muscle power in the lower limbs and were unable to walk unaided. One patient with 140° kyphoscoliotic deformity with grade 3 muscle power had post-polio residual paralysis (PPRP) in addition. All patients were subjected to thorough anterior spinal decompression through transthoracic, transpleural thoracotomy from the left side.

**Results:**

In six of the eight patients, the spine at the site of deformity being very rigid, the deformity could not be corrected and the intervertebral gap was bridged with appropriate autogenous tricortical cortico cancelluous bone graft. In one patient (case 4), the kyphotic deformity could be corrected by 50%. In one patient with 140° kyphosis and PPRP, the gap after the decompression of cord, could not be bridged with bone graft and was given a custom made, well molded plastic black shell to wear while walking and, in particular, while traveling in a vehicle. In all seven patients, bone grafts took six months for bridging the intervertebral gaps. All patients recovered to grade 4 muscle power 6–12 months after surgery.

**Conclusion:**

In adolescents with healed caries of dorsal spine with acute kyphosis and paraplegia, the treatment of choice is anterior surgical decompression of the cord and bridging the gap thus created with bone graft.

## INTRODUCTION

Sorrel-Dejerine^1^,^2^ was the first to draw attention to two types of paraplegia associated with caries of the dorsal spine depending on the time of onset:^1^ early onset in acute infective phase and^2^ late onset occurring many years after the infection. The paraplegia of early onset is caused either because of edema or compression of the cord by an abscess, granulation tissue, which would invariably recover neurologically as inflammation regresses. Paraplegia of late onset, was considered to be due to pachymeningitis, which showed poor or no neural recovery. Butler^3^ and Seddon,^4^ independently studying their cases with paraplegia, agreed with Sorrel-Dejerine's distinction between paraplegia of early onset and late onset. All their cases with paraplegia of early onset did not recover spontaneously (one-fourth of their patients did not recover), and about half of their patients with paraplegia coming on late made a good recovery. So, they disagreed in part with her views on pathology and completely disagreed with her clear-cut statements on prognosis. Seddon^5^ in particular stated that the pathogenesis in paraplegia of late onset was uncertain and the prognosis was doubtful.

The author presents a study of eight adolescent patients with paraplegia of late onset associated with acute kyphotic deformity of dorsal spine to find the cause of paraplegia, the final neurological outcome following anterior decompression and its prevention.

## MATERIALS AND METHODS

Between 1970 and 2001, eight adolescent patients between the ages of 13 and 15 years (mean age 14.4 years) in fairly good health, coming from low-income group of rural community, presented with late onset of paraplegia and acute kyphotic deformity of dorsal spine. There were six males and two females. All of them had tuberculosis infection of dorsal spine between the ages of six and eight years, with an average age of onset being 6.9 years. Six of them had irregular antituberculous treatment, and in two others, there was no definite history of having had treatment. Four of the six patients stated that they had streptomycin injections and oral isoniazid (INH) and paramino salicylic acid (PAS). Two others seemed to have had oral medication but could not mention what exact medication they had. None of the eight patients had any neurological deficit on initial presentation.

Clinically all eight patients had severe kyphotic deformity in dorsal spine. Seven patients had kyphotic deformity while one (case-8) had kyphoscolosis deformity of dorsal spine with post-polio residual paralysis (PPRP) [Table 1].

**Table 1:** Showing clinical details of the patients

S no.	Sex	Age	Number of vertebral bodies involved	Angle of kyphosis°	The bone grafts used
1	F	15	D7 D8	90	Iliac tricortical graft
2	M	15	D4 D5 D6	70	Fibular strut graft
3	M	15	D3 D4	60	Iliac tricortical graft
4	M	15	D11 D12	70	Iliac tricortical graft
5	M	14	D4 D5 D6	65	Fibular strut graft
6	F	13	D1 D2 D3	75	Fibular strut graft
7	M	13	D1 D2 D3	75	Fibular strut graft
8	M	15	D5 D6 D7	140	No bone graft used

**Figure 1:**
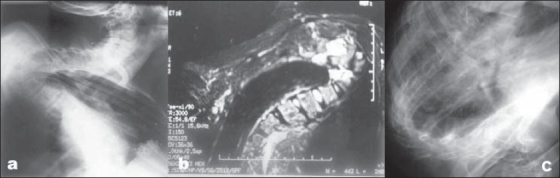
(a) Preoperative plain skiagram (b) Midsagittal T2-weighted MRI image of dorsal spine showing severe kyphoscoliosis with internal gibbus at the apex of the curve. (c) Postoperative skiagram taken six months after surgery shows gap grafting and correction of kyphosis.

On presentation, all patients had upper motor neuron type of paraplegia with exaggerated ankle and knee jerks and positive Babinski's sign. Two patients had MRC grade 0 motor power, four had grade 2 power in lower limb muscles, and two others had grade 3 power in lower limb muscles and were unable to walk unaided. All hematological and other laboratory investigations were normal. None of them was subjected to pulmonary function tests, nor anesthetists asked for them. Plain skiagrams of the dorsal spine in all patients showed severe kyphotic deformity of spine as measured by modified Konstam's method. Kyphosis averaged 80°, ranging from 60° to 140° [Table 1]. Three vertebral bodies were affected in six patients; and two vertebral bodies were affected in the rest. The upper dorsal spine was involved in two, mid-dorsal in five, and lower dorsal in one. There was no evidence of active disease at the level of kyphotic deformity in all patients. There was internal gibbus in all patients with narrowing the spinal canal at the kyphotic deformity. One patient (case 8) had 140° kyphoscoliotic deformity in middorsal spine, with PPRP [Figure 1a]. Two patients (cases 7 and 8) were subjected to magnetic resonance imaging. In mid sagittal, MRI of upper dorsal spine in case 7, the C_7_ vertebral body was tilted forward over fused posterior cartilaginous remains of affected D_1_, D_2_, D_3_ and D_4_ vertebral bodies, causing severe compression of the cord at D_4_ body [Figure 2a]. In mid sagittal MRI of case 8, the gibbus could be seen in the upper dorsal region, but the cord compression could not be appreciated due to kyphoscoliosis of the dorsal spine [Figure 1b].

All patients were subjected to anterior spinal decompression via transthoracic, transpleural thoracotomy from left side.^6^ In six patients, through parascapular incision, the level of cord compression could be exposed after transthoracic, transpleural approach after excising the second or third left rib. In the remaining two patients, through left oblique transthoracic, transpleural approach, the appropriate rib corresponding to the kyphotic deformity in the mid axillary region was excised to expose the level of spinal compression. The internal gibbus in seventh patients could be exposed and excised thoroughly after excising one vertebral body above and another fully or partially below the level of gibbus. The exposed cord in all seven cases was seen pulsating. The intervertebral gap was bridged with appropriate autogenous tricortical bone graft. In six patients, the kyphotic deformity was found to be rigid and could not be corrected [Figure 2a–f]. In one patient (case-8) with lesion at D_11_, D_12_, the 70° kyphotic deformity after excising internal gibbus could be corrected to about 50% before bridging the vertebral gap with bone graft [Figure 3a-b]. In case 8 with 140° kyphoscoliotic deformity associated with PPRP, the internal gibbus could be excised thoroughly after excising one vertebral body each above and below completely and another partially below the internal gibbus. The exposed cord was seen and felt pulsating. The intervertebral gap could not be bridged with bone graft, and posterior spinal fusion could not be performed because of severe kyphotic deformity associated with rigid scoliosis. A custom-made plastic posterior shell was provided to be worn during ambulation. There was no evidence of active infection intraoperatively at the level of deformity and internal gibbus in all patients.

**Figure 2:**
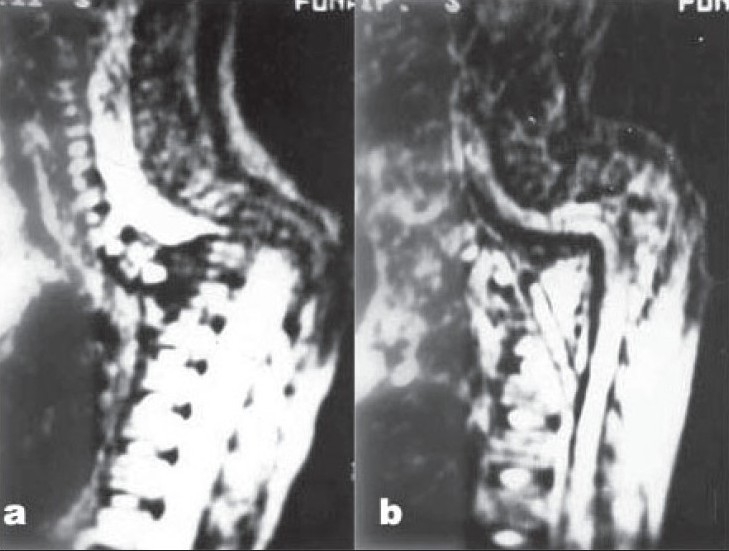
(a) Mid sagittal T2 WI MRI of upper dorsal spine showing forward tilt of the C7 body over fused posterior cartilaginous remains of D1-D3 vertebral bodies and D4 vertebral body causing severe compression of cord at D4 body. (b) Postoperative mid sagittal T2 WI MRI showing through decompression of the cord and fibular graft bridging the gap. note the organized soft tissue between graft and cord.

**Figure 2:**
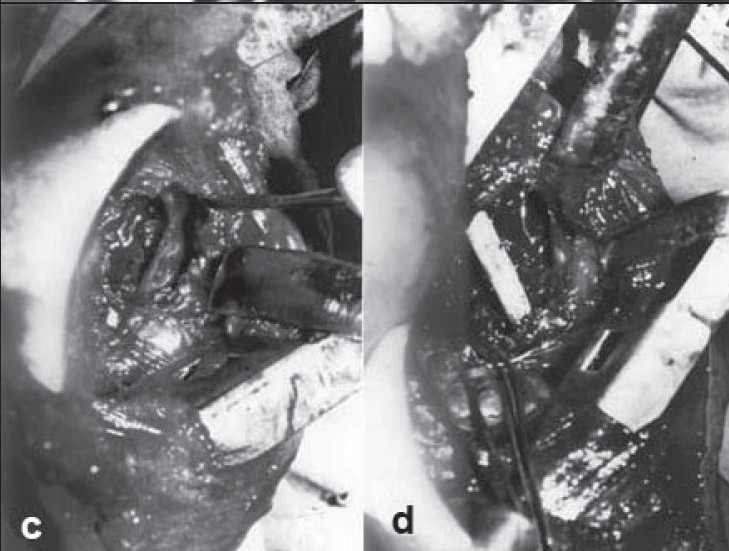
(c and d) Intraoperative photographs showing cord after decompression and fibular graft bridging the gap. Note the kink in cord due to pressure.

**Figure 2:**
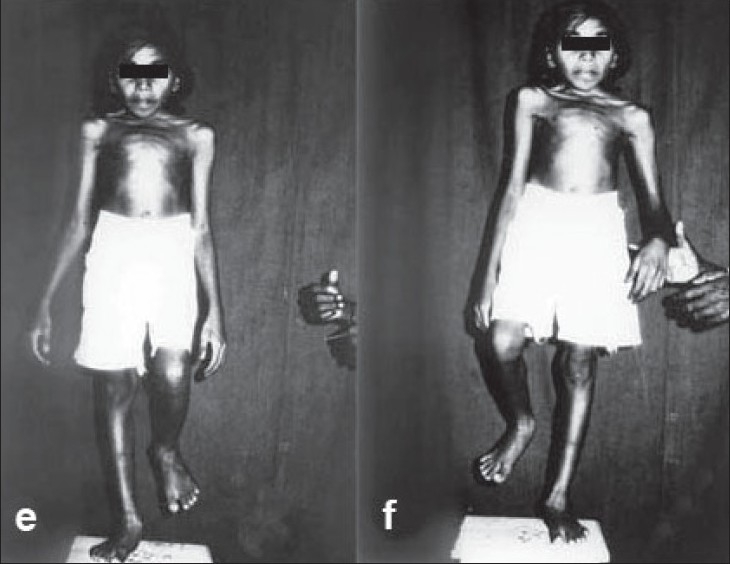
(e and f) Clinical photograph showing full recovery from paraplegia after surgery. Note the patient (case7) standing on each leg without support.

**Figure 3:**
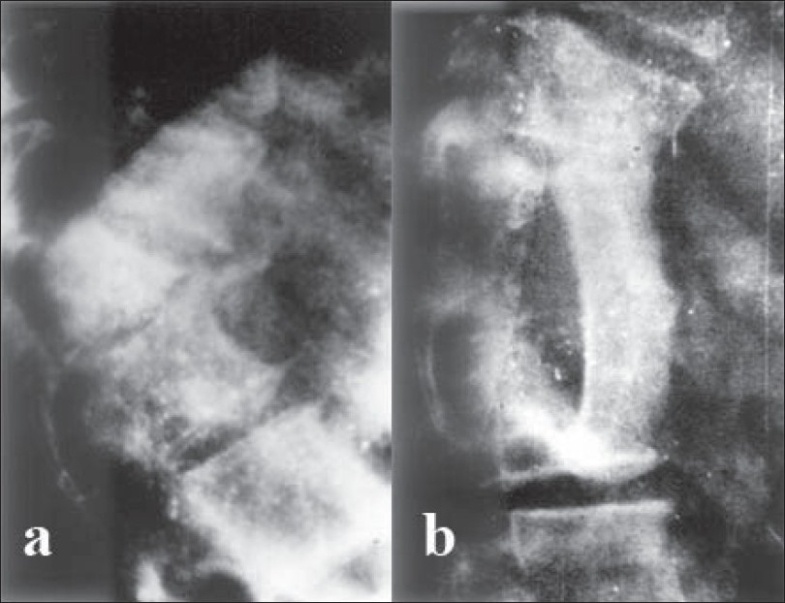
(a) Preoperative skiagram of dorsal lumbar spine showing acute kyphotic deformity with locked wedge shaped internal gibbus, as the cause for spinal stenosis. (b) Postoperative skiagram showing thorough excision of the internal gibbus and iliac bone graft bridging the gap. Note the correction of kyphotic deformity.

**Figure 4:**
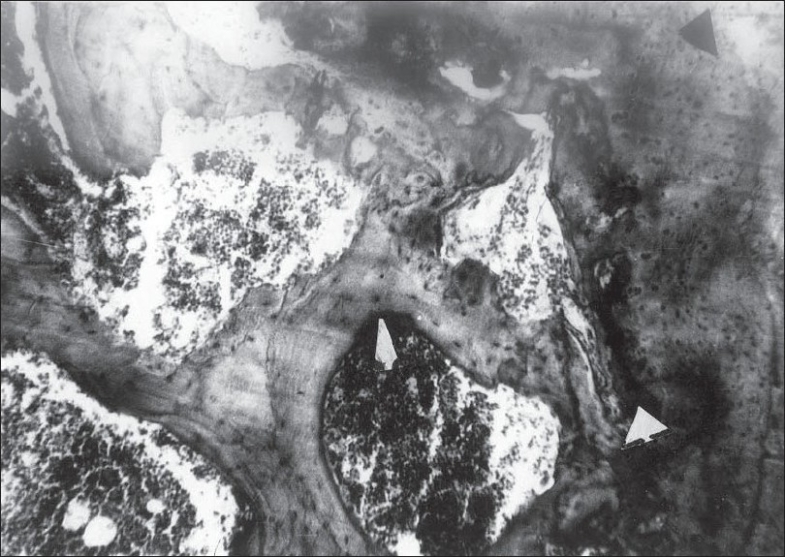
Photomicrograph of internal gibbus showing areas of uncalcified cartilage (shown by black pointer), calcified cartilage (shown by wide white pointer) and well-formed bone with blood space (shown by thin white pointer). (H&E; ×60)

None of the patients had any intraoperative and postoperative complications including chest infection. Postoperative recovery was uneventful, and in two other patients, there was superficial infection resulting in delayed wound healing. None of the patients was given antituberculous treatment because there was no intraoperative evidence of infection, the histopathological examination of excised bits of internal gibbus, showed no evidence of active infection. It showed well formed bone with blood filled sinusoidal spaces and calcified and uncalcified hyaline cartilage [Figure 4].

## RESULTS

All patients were ambulated when they gained grade 4 power in lower limb muscles. Two patients with grade 3 power recovered in six months to grade 4 power, four patients with grade 2 power recovered to grade 4 in about eight months, and two patients with grade-0 power recovered to grade 4 power in about a year [Figure 2e-f]. The follow-up was possible for about six months after recovery in all six patients.

Postoperative skiagrams of all patients including case 8 with 140° kyphotic deformity did not show any change in kyphotic deformity. All patients were treated postoperatively with neurotrophic medicines, such as pridoxine and methyl cobalamine.

## DISCUSSION

Capener^7^ was the first to report paraplegia in healed caries of the dorsal spine with kyphosis due to the stretch of the cord over a bony ridge in the spinal canal. Seddon^5^ was the first to state, “Paraplegia of late onset may however occur in the absence of reactivation of tuberculous focus…. This spine is invariably and grossly angulated; the affected vertebral bodies may be solidly fused. The disease is cured yet patient becomes paraplegic.” He suspected acute bony ridge in the floor of spinal canal following severe kyphotic deformity the cause for stretching of the cord. This view was supported by others.^8^–^10^ Not realizing that the posterior cartilaginous remains of the destroyed vertebral bodies would grow backward, Hodgson and Yau^11^ postulated that the essential factor in the cause of paraplegia of late onset was increasing kyphosis that gradually squeezes the healing bone backward on to the cord. Rajasekaran^12^ was also of the same view that in some children the remnants of destroyed vertebral bodies would be retropulsed. However, he did not state the effect of retropulsion of the remnants of destroyed vertebral bodies on the cord.

The regeneration of partially destroyed vertebral bodies during growth is documented only when the anterior parts of vertebral bodies are present. Rajasekaran^12^ observed, in a study of 28 children under the age of 15 years, the anterior growth of the affected vertebral bodies and decrease in the angle of kyphotic deformity in 10 cases during ambulatory treatment. He thought that the increase in kyphotic deformity was “influenced by severity of pretreatment angle of the deformity, the extent of initial vertebral loss and importantly by an instability of more than 2.” The author thinks that the increase of kyphotic deformity depends on the pretreatment destruction of the anterior parts of the diseased vertebral bodies and greater remains of their posterior bony cartilaginous masses.

**Figure 5:**
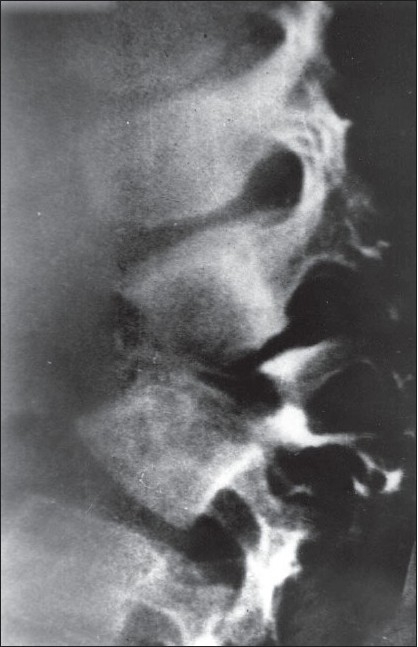
Plain lateral skiagrams of dorso-lumbar spine in a child with healed caries showing posterior bony cartilaginous remains of destroyed vertebral bodies locked in between and blocked anteriorly by tilt of upper normal over lower normal vertebral body

Cleveland *et al*,^13^ after observing 18 of their patients for 21 years, found that the growth of fusion mass occurred in both sagittal and coronal planes. however, they did not specify whether the fused mass could be due to fusion of the affected vertebral bodies with anterior remains or due to the fusion of only the posterior remains of the affected vertebral bodies. However, so far, there is no specific documentation regarding the nature of the growth of healed and fused posterior bony cartilaginous remains of destroyed vertebral bodies in children locked in between and blocked anteriorly by the tilt of the upper normal over lower normal vertebral body. By observing the skiagrams of the spine of the children with healed caries with posterior bony cartilaginous vertebral remains of destroyed vertebral bodies locked in between and blocked anteriorly by the tilt of the upper normal over the lower normal vertebral body [Figure 5] and the skiagrams of adolescents with paraplegia having fused wedge-shaped posterior remains of the destroyed vertebral bodies locked and blocked anteriorly by the tilt of upper normal over lower normal vertebral body [Figure 3a], the author came to the conclusion that the posterior bony cartilaginous remains of destroyed vertebral bodies fused to form bony wedge-shaped cartilaginous mass in children. This wedge-shaped mass during the last spurt of growth during adolescence would grow upward and downward, as any normal vertebral body, to increase the kyphotic deformity. The activity of kyphotic deformity depends on the greater wedge-shaped mass, which in turn depends on the bulk of posterior bony cartilaginous remains of destroyed vertebral bodies. Besides, this wedge-shaped mass being blocked anteriorly takes retrograde growth into spinal canal to form a hump-like internal gibbus, causing narrowing of the spinal canal and thus pressure over the front of cord to result in paraplegia.

The author is of the opinion that paraplegia of late onset should be treated by anterior decompression after thorough excision of internal gibbus causing compression of the cord and by bridging the gap with appropriate autogenous bone graft.

Two articles on Pott's paraplegia of late onset were published^9^,^10^. Bilsel *et al*.^9^ stated that all their eight cases developed neurological deficit following the formation of sharp kyphosis and treated by anterior decompression and bone grafting without correcting the kyphotic deformity. But, they did not specify whether they treated the late-onset paraplegia with or without active disease in adolescents. Hau *et al*.^10^ reported 22 cases of Pott's paraplegia of late onset. Out of them, eight cases had late-onset paraplegia with healed disease and the rest 14 cases had an active disease. They treated all cases with active disease by anterior decompression because they thought the excision of the cause for pressure on cord was technically simpler because of the presence of abscess cavity often connected to the front of the spinal cord and the tissue of internal kyphosis was softer. In all their cases of late paraplegia with healed disease, the cord was decompressed through anterolateral approach after widening the intervertebral foramen by tracking the corresponding segmental nerve root.

Their results of recovery following anterolateral decompression of eight cases with late paraplegia are listed as follows:

1. One patient with severe paraplegia and two with minimal neurological deficit recovered fully.
2. Out of four cases with moderate paraplegia, two were unchanged and two improved slightly.
3. One with moderate signs of paraplegia became totally paralyzed.

In view of such an outcome following anterolateral decompression in cases of paraplegia with healed caries spine, they thought “the patient with hard ridge compression and only mild to moderate paraplegia should perhaps be treated by stabilization only, to prevent further progression of the paraplegia, and that the decompression should be reserved for those with severe paralysis.” They finally concluded that in the cases with paraplegia due to healed disease with bony ridge, the results of recovery following surgery were not rewarding.

The author firmly believes that through anterolateral decompression, thorough decompression of cord cannot be performed, as the part of internal gibbus on the opposite side of surgery causing pressure on the cord cannot be reached and excised. In caries spine vertebral body is mostly involved, and in Pott's paraplegia, decompression should be performed from the anterior side.^8^,^14^ As such, in his opinion, by thorough anterior approach only, the entire internal gibbus formed in adolescence by the fusion of healed childhood posterior remains of destroyed vertebral bodies causing pressure on the cord could be thoroughly exposed and excised after excising one vertebral body above and another fully or partially below the level of internal gibbus.

As paraplegia of late onset with healed childhood dorsal caries spine is a very difficult problem, it could be prevented in children with active dorsal caries spine if diagnosed early and treated effectively, preventing the destruction of vertebral bodies and development of kyphotic deformity^14^,^15^ But, children when present with active caries spine and kyphotic deformity with or without paraplegia should be subjected to anterior surgical exploration for thorough excision of diseased vertebral bodies until the cord is well exposed and kyphus is corrected surgically.^16^ The spinal gap thus formed is bridged with appropriate autogenous bone graft.^8^,^17^
